# Supplementary material for: Mutual interaction between visual homeostatic plasticity and sleep in adult humans
Source: eLife. 2022 Aug 16;11:e70633. doi: 10.7554/eLife.70633 (PMC9417418; doi:10.7554/eLife.70633)
Supplement: Supplementary file 1. [file elife-70633-supp1.docx]

**Table - Sleep Macrostructural Parameters: MDnight and Cnight descriptive statistics**

|  |  |  |  |  |  |  |  | **Spearman's corr** | | | | | |
| --- | --- | --- | --- | --- | --- | --- | --- | --- | --- | --- | --- | --- | --- |
|  | **MDnight** | | **Cnight** | | **wilcoxon paired**  **comparison** | |  | **DI before sleep** | | | **DI after sleep** | | |
|  | median | iqr | median | iqr | p | p_fdr |  | rho | p | p_fdr | rho | p | p_fdr |
| **sleep latency (min)** | 7 | 9 | 9 | 10 | 0.62 | 0.86 |  | 0.14 | 0.62 | 0.70 | 0.15 | 0.59 | 0.91 |
| **N2 (min)** | 43 | 14 | 43 | 22 | 0.88 | 1.00 |  | 0.06 | 0.83 | 0.83 | -0.26 | 0.35 | 0.91 |
| **N3 (min)** | 56 | 23 | 53 | 26 | 0.65 | 0.86 |  | -0.20 | 0.47 | 0.70 | -0.01 | 0.96 | 0.96 |
| **REM (min)** | 0 | 8 | 4 | 7 | 0.47 | 0.86 |  | -0.57 | 0.03 | 0.21 | 0.12 | 0.66 | 0.91 |
| **WASO (min)** | 6 | 4 | 6 | 5 | 0.28 | 0.86 |  | -0.22 | 0.43 | 0.70 | 0.12 | 0.68 | 0.91 |
| **REM latency (min)** | 92 | 44 | 84 | 30 | 0.44 | 0.86 |  | 0.31 | 0.56 | 0.70 | -0.09 | 0.92 | 0.96 |
| **shift phase (1/min)** | 0.13 | 0.13 | 0.14 | 0.03 | 0.28 | 0.86 |  | -0.23 | 0.42 | 0.70 | -0.41 | 0.13 | 0.50 |
| **sleep fragmentation (1/min)** | 0.02 | 0.03 | 0.02 | 0.02 | 1.00 | 1.00 |  | -0.18 | 0.53 | 0.70 | -0.59 | 0.02 | 0.19 |

iqr= interquartiles range; fdr=FDR correction.
