# Supplementary material for: Mutual interaction between visual homeostatic plasticity and sleep in adult humans
Source: eLife. 2022 Aug 16;11:e70633. doi: 10.7554/eLife.70633 (PMC9417418; doi:10.7554/eLife.70633)
Supplement: Supplementary file 2. [file elife-70633-supp2.docx]

**Table: features comparison between conditions and correlations with plasticity indices for the relevant ROIs**

|  | main effect (wilcoxon test) | | correlations with plasticity indices (Spearman's correlation) | | | | | |
| --- | --- | --- | --- | --- | --- | --- | --- | --- |
|  | **MDnight vs MDc** |  | **DI before** |  |  | **DI after** |  |  |
| ROI/ EEG sleep feature | p-value | p-value  (fdr) | rho | p-value | p-value  (fdr) | rho | p-value | p-value  (fdr) |
| sensory-motor / slope+ | 0.76 | 0.87 | -0.13 | 0.65 | 0.75 | -0.05 | 0.86 | 0.92 |
| sensory-motor / NP amp | 1.00 | 1.00 | -0.23 | 0.42 | 0.67 | -0.05 | 0.86 | 0.92 |
| sensory-motor / SSO sigma baseline | 0.33 | 0.75 | -0.21 | 0.46 | 0.67 | -0.23 | 0.41 | 0.86 |
| sensory-motor / spindle power | 0.21 | 0.75 | -0.09 | 0.75 | 0.75 | -0.30 | 0.27 | 0.86 |
| sensory-motor / SSO density | 0.02 | 0.29 | 0.12 | 0.67 | 0.75 | -0.02 | 0.94 | 0.94 |
| sensory-motor / spindle density | 0.68 | 0.87 | 0.12 | 0.68 | 0.75 | -0.38 | 0.16 | 0.86 |
| sensory-motor / SWA | 0.52 | 0.84 | -0.25 | 0.37 | 0.65 | 0.10 | 0.73 | 0.92 |
| sensory-motor / sigma power | 0.21 | 0.75 | -0.27 | 0.33 | 0.65 | -0.16 | 0.58 | 0.92 |
| occipital / slope+ | 0.76 | 0.87 | **-0.64** | **0.01** | **0.04** | -0.01 | 0.98 | 0.98 |
| occipital / NP amp | 0.68 | 0.87 | -0.56 | 0.03 | 0.09 | 0.14 | 0.63 | 0.98 |
| occipital / SSO sigma baseline | 0.39 | 0.78 | **-0.70** | **0.00** | **0.04** | 0.09 | 0.75 | 0.98 |
| occipital / spindle power | 0.98 | 1.00 | **-0.66** | **0.01** | **0.04** | -0.07 | 0.80 | 0.98 |
| occipital / SSO density | 0.05 | 0.38 | **-0.66** | **0.01** | **0.04** | -0.35 | 0.20 | 0.98 |
| occipital / spindle density | 0.52 | 0.84 | 0.10 | 0.73 | 0.75 | -0.03 | 0.91 | 0.98 |
| occipital / SWA | 0.28 | 0.75 | -0.42 | 0.12 | 0.28 | 0.14 | 0.63 | 0.98 |
| occipital / sigma power | 0.33 | 0.75 | **-0.72** | **0.00** | **0.04** | 0.12 | 0.67 | 0.98 |
| prefrontal / slope+ | 0.85 | 0.90 |  |  |  | 0.20 | 0.48 | 0.86 |
| prefrontal / NP amp | 0.11 | 0.61 |  |  |  | -0.08 | 0.79 | 0.92 |
| prefrontal / SSO sigma baseline | 0.64 | 0.84 |  |  |  | -0.25 | 0.37 | 0.86 |
| prefrontal / spindle power | 0.45 | 0.84 |  |  |  | -0.24 | 0.38 | 0.86 |
| prefrontal / SSO density | 0.23 | 0.61 |  |  |  | -0.21 | 0.45 | 0.86 |
| prefrontal / spindle density | 0.68 | 0.84 |  |  |  | **-0.74** | **0.00** | **0.04** |
| prefrontal / SWA | 0.52 | 0.84 |  |  |  | 0.40 | 0.14 | 0.86 |
| prefrontal / sigma power | 0.23 | 0.61 |  |  |  | -0.06 | 0.84 | 0.92 |
